# Supplementary material for: Microenvironmental Factors in Oral Cavity Squamous Cell Carcinoma Undergoing Surgery: Correlation with Diffusion Kurtosis Imaging and Dynamic Contrast-Enhanced MRI
Source: Cancers (Basel). 2022 Dec 20;15(1):15. doi: 10.3390/cancers15010015 (PMC9817509; doi:10.3390/cancers15010015)
Supplement: Supplementary file 1 [file cancers-15-00015-s001.zip › Supplementary Materials.zip!/Supplementary Materials.pdf]

Content:

Tables

- ✓ **Supplementary Table S1:** Results of Shapiro-Wilk Normality Test from DCE-MRI Parameters
- ✓ **Supplementary Table S2:** Results of Shapiro-Wilk Normality Test from DKI-derived Parameters
- ✓ **Supplementary Table S3:** Results of Shapiro-Wilk Normality Test from numerical pathological parameters
- ✓ **Supplementary Table S4:** *p*-values derived from Wruskal-Wallis Test to assess the effect of the pathological factors on DCE-MRI and DKI parameters

Supplementary Table S1: Results of Shapiro-Wilk Normality Test of DCE-MRI Parameters

Shapiro-Wilk Test	Test Statistic	p-value	Normality
Median of K^{trans}	0,802	0,000	0
IQR of K^{trans}	0,858	0,000	0
P10 of K^{trans}	0,825	0,000	0
P25 of K^{trans}	0,804	0,000	0
P75 of K^{trans}	0,899	0,002	0
P90 of K^{trans}	0,910	0,004	0
Skewness of K^{trans}	0,925	0,013	0
Kurtosis of K^{trans}	0,665	0,000	0
Mean of K^{trans}	0,913	0,005	0
**Std of K^{trans}**	0,954	0,114	**1**
Energy of K^{trans}	0,865	0,000	0
Entropy of K^{trans}	0,929	0,017	0
Median of K_{ep}	0,879	0,001	0
IQR of K_{ep}	0,898	0,002	0
P10 of K_{ep}	0,867	0,000	0
P25 of K_{ep}	0,874	0,000	0
P75 of K_{ep}	0,927	0,015	0
P90 of K_{ep}	0,864	0,000	0
Skewness of K_{ep}	0,896	0,002	0
Kurtosis of K_{ep}	0,689	0,000	0
Mean of K_{ep}	0,790	0,000	0
Std of K_{ep}	0,780	0,000	0
Energy of K_{ep}	0,928	0,016	0
**Entropy of K_{ep}**	0,985	0,874	**1**
Median of v_e	0,935	0,026	0
**IQR of v_e**	0,968	0,327	**1**
**P10 of v_e**	0,948	0,069	**1**
P25 of v_e	0,901	0,002	0
P75 of v_e	0,850	0,000	0
P90 of v_e	0,552	0,000	0
Skewness of v_e	0,932	0,021	0
Kurtosis of v_e	0,607	0,000	0
**Mean of v_e**	0,964	0,244	**1**
**Std of v_e**	0,969	0,340	**1**
Energy of v_e	0,601	0,000	0
Entropy of v_e	0,763	0,000	0

Abbreviations: Std, standard deviation; IQR, Inter-quartile range. Variables normally distributed are in **bold**.

Supplementary Table S2: Results of Shapiro-Wilk Normality Test of DKI-derived Parameters

Shapiro-Wilk Test	Test Statistic	p-value	Normality
Median of D	0,932	0,026	0
IQR of D	0,933	0,028	0
**P10 of D**	0,944	0,063	**1**
P25 of D	0,900	0,003	0
**P75 of D**	0,962	0,242	**1**
**P90 of D**	0,947	0,079	**1**
**Skewness of D**	0,980	0,719	**1**
Kurtosis of D	0,893	0,002	0
**Mean of D**	0,944	0,060	**1**
**Std of D**	0,943	0,057	**1**
Energy of D	0,791	0,000	0
Entropy of D	0,891	0,002	0
**Median of K**	0,988	0,953	**1**
**IQR of K**	0,963	0,246	**1**
P10 of K	0,898	0,003	0
**P25 of K**	0,983	0,845	**1**
**P75 of K**	0,956	0,147	**1**
**P90 of K**	0,950	0,097	**1**
Skewness of K	0,874	0,001	0
Kurtosis of K	0,606	0,000	0
**Mean of K**	0,970	0,397	**1**
**Std of K**	0,966	0,317	**1**
Energy of K	0,860	0,000	0
Entropy of K	0,933	0,029	0

Abbreviations: Std, standard deviation; IQR, Inter-quartile range. Variables normally distributed are in **bold**.

Supplementary Table S3: Results of Shapiro-Wilk Normality Test from numerical pathological parameters

Shapiro-Wilk Test	Test Statistic	p-value	Normality
TSR	0,939	0,042	0
TILs	0,841	0,000	0

Abbreviations: TSR, Tumor stroma ratio; TIL, tumor-infiltrating lymphocytes.

Supplementary Table S4: *p*-values derived from the Wruskal-Wallis test to assess the effect of the pathological factors on DCE-MRI and DKI parameters (only statistically significant results are reported)

	Kurtosis of K_{ep}												
Perineural infiltration	0,044												
	Skewness of K												
CD3/20 prominence	0,048												
	Energy of K^{trans}												
CD4/8 prominence	0,019												
	Energy of D	Entropy of D											
Keratinization	0,018	0,029											
	Median of D	P25 of D	P90 of D	Mean of D									
Inflammatory infiltrate	0,042	0,033	0,047	0,013									
	IQR of K_{ep}	P75 of K_{ep}	P90 of K_{ep}	Energy of K_{ep}	Entropy of K_{ep}	IQR of v_e	Std of v_e						
Grading	0,024	0,035	0,041	0,034	0,023	0,027	0,005						
	P25 of K^{trans}	Median of K_{ep}	P10 of K_{ep}	P25 of K_{ep}	P75 of K_{ep}	P90 of K_{ep}	Mean of K_{ep}	Entropy of K_{ep}	P10 of v_e	P25 of v_e	Std of v_e	Skewness of K	Kurtosis of K
Desmoplastic reaction	0,031	0,018	0,011	0,026	0,035	0,017	0,036	0,019	0,004	0,047	0,008	0,015	0,010
